# Nafamostat mesilate, a nuclear factor kappa B inhibitor, enhances the antitumor action of radiotherapy on gallbladder cancer cells

**DOI:** 10.1371/journal.pone.0257019

**Published:** 2021-09-02

**Authors:** Naoki Takada, Hiroshi Sugano, Yoshihiro Shirai, Nobuhiro Saito, Ryoga Hamura, Tomohiko Taniai, Tadashi Uwagawa, Katsuhiko Yanaga, Toru Ikegami, Toya Ohashi, Ken Eto

**Affiliations:** 1 Department of Surgery, The Jikei University School of Medicine, Tokyo, Japan; 2 Division of Gene Therapy, Research Center for Medical Science, The Jikei University School of Medicine, Tokyo, Japan; 3 Division of Clinical Oncology and Hematology, Department of Internal Medicine, The Jikei University School of Medicine, Tokyo, Japan; Duke University School of Medicine, UNITED STATES

## Abstract

Nuclear factor kappa B (NF-κB) is a transcriptional factor that can be activated by radiotherapy and chemotherapy. The synthetic protease inhibitor nafamostat mesilate (NM) inhibits NF-κB activity and exerts antitumor actions in various types of cancer. In the present study, we hypothesized that NM might enhance the antitumor action of radiotherapy on gallbladder cancer (GBC) cells by inhibiting radiation-induced NF-κB activity. Thus, we investigated the correlation between radiotherapy and NF-κB activity in GBC cells. We assessed the *in vitro* effects of radiotherapy with or without NM on NF-κB activity, apoptosis of GBC cells (NOZ and OCUG-1), induction of apoptotic cascade, cell cycle progression, and viability of GBC cells using four treatment groups: 1) radiation (5 Gy) alone; 2) NM (80 μg/mL and 40 μg/mL, respectively) alone; 3) combination (radiation and NM); and 4) vehicle (control). The same experiments were performed *in vivo* using a xenograft GBC mouse model. *In vitro*, NM inhibited radiation-induced NF-κB activity. Combination treatment significantly attenuated cell viability and increased cell apoptosis and G2/M phase cell cycle arrest compared with those in the other groups for NOZ and OCUG-1 cells. Moreover, combination treatment upregulated the expression of apoptotic proteins compared with that after the other treatments. *In vivo*, NM improved the antitumor action of radiation and increased the population of Ki-67-positive cells. Overall, NM enhanced the antitumor action of radiotherapy on GBC cells by suppressing radiation-induced NF-κB activity. Thus, the combination of radiotherapy and NM may be useful for the treatment of locally advanced unresectable GBC.

## Introduction

Gallbladder cancer (GBC) is one of the gastrointestinal cancers with a poor prognosis. According to the National Cancer Database, the five-year survival of patients with GBCs recorded between 1989 to 1996 was 50% and 2% for stages I and IV, respectively [1]. The standard treatments for GBC are surgery, chemotherapy, and radiotherapy. Surgical resection is the only option to cure GBC. However, the resectability rate of GBC is limited because of the difficulty in diagnosis at the early stage [2]. The median survival time of unresectable GBC is 11.7 months with chemotherapy using gemcitabine with cisplatin being the first-line treatment [3]. Radiotherapy and chemoradiotherapy have been reported as neoadjuvant therapies for locally advanced unresectable GBC [4–7]. However, the effect of radiotherapy is not satisfactory due to radio-resistance of cells. Although the molecular mechanism of radio-resistance is not clear, there are a few reports on the correlation between GBC and radiotherapy [8]. Hence, novel treatment strategies need to be developed to improve the radiosensitivity of GBC.

Nuclear factor kappa B (NF-κB) is a transcriptional factor involved in numerous physiological phenomena, such as inflammatory and immune responses, cell proliferation, and cell apoptosis [9, 10]. Additionally, NF-κB plays a central role in the progression, carcinogenesis, and treatment (e.g. chemotherapy and radiotherapy) resistance of various types of cancer [10–12]. NF-κB is formed by heterodimers of p50 and p65 and exists in an inactive form in the cytoplasm by binding with inhibitor of kappa B (IκB). When IκB kinase complex is activated by stimulation such as chemotherapy and radiotherapy, IκB is phosphorylated and activated NF-κB moves to the nucleus. Activated NF-κB causes the transcription of genes, such as those of antiapoptotic proteins, interleukins, and adhesion molecules, resulting in treatment resistance [10, 13–15].

We previously reported that the synthetic protease inhibitor nafamostat mesilate (NM), commonly used in Japan for pancreatitis and disseminated intravascular coagulation (DIC), suppresses NF-κB activity and induces antitumor actions on pancreatic cancer [16]. Moreover, we showed that NM exerts antitumor action by suppressing radiation-induced NF-κB activity in a mouse model induced by pancreatic and colon cancer cell lines [17, 18]. Hence, in this study, we first investigated the correlation between radiotherapy and NF-κB activity in GBC cells. We also evaluated the role of NM in inhibiting radiation-induced NF-κB activity and enhancing the antitumor action of radiotherapy on GBC cells.

## Materials and methods

### Cell cultures

The human GBC cell lines NOZ and OCUG-1 were purchased from the Health Science Research Resources Bank (Osaka, Japan). The cells were cultured in Dulbecco’s modified Eagle’s medium with L-glutamine (Wako Pure Chemical Industries, Ltd., Osaka, Japan) supplemented with 1% penicillin/ streptomycin (Gibco BRL, Grand Island, NY, USA) and 10% fetal bovine serum (Gibco BRL). The cells were incubated with 5% carbon dioxide at 37°C.

### Reagent

Nafamostat mesilate was received from Torii Pharmaceutical Co., Ltd. (Tokyo, Japan) and was dissolved in distilled water (DW) to 5 mg/mL and then frozen at -20°C.

### Ionizing radiation

In all experiments, the cells and the subcutaneous tumors were directly irradiated with 5 Gy using an X-ray irradiator MBR-1520R-3 (HITACHI Engineering & Services Co., Ltd., Tokyo, Japan). *In vivo*, during irradiation, the mice were covered with a protector HAGOROMO (Maeda Co., Tokyo, Japan), except at the subcutaneous tumor in the right flank.

### Antibodies

Monoclonal antibodies specific to phosphorylated IκBα, IκBα, cleaved caspase-9, cleaved caspase-3, and cleaved PARP were obtained from Cell Signaling Technology (Beverly, MA, USA). Monoclonal anti-β-actin antibody was obtained from Sigma Aldrich (St. Louis, MO, USA). Monoclonal anti-Ki-67 antibody was obtained from Dako (Glostrup, Denmark).

### *In vitro* experimental groups

NOZ and OUCG-1 cells were divided into the following treatment groups: (1) NM group, GBC cells treated with NM alone (80 and 40 μg/mL, respectively); (2) radiation group, GBC cells treated with radiation alone; (3) combination group, GBC cells treated with both NM (80 and 40 μg/mL, respectively) and radiation; and (4) control group, GBC cells treated with vehicle only. In all *in vitro* experiments, the cells in the radiation and combination groups received radiation of 5 Gy. In the combination group, the cells received radiation at 3 h after NM administration.

### Cell proliferation assay

NOZ and OCUG-1 cells were seeded into 96-well plates (1 × 10^3^ and 2 × 10^3^ cells per well, respectively) and subjected to each treatment regimen for 96 h. Cell proliferation was measured using the Premix WST-1 Cell Proliferation Assay (Takara Bio Inc., Shiga, Japan).

### Colony forming assay

NOZ and OCUG-1 cells were seeded in 6-well plates (2 × 10^3^ and 5 × 10^3^ cells per well, respectively) and subjected to each treatment regimen. GBC cells were cultured for 12 days to allow colony formation. After culturing, the colonies were washed PBS and fixed with 80% ethanol for 5 minutes at room temperature. The colonies were stained with 10% Giemsa solution (Wako, Osaka, Japan) for 10 minutes at room temperature. After staining, the colonies were washed with PBS and dried. The stained colonies were counted and surviving fraction were calculated.

### Quantitative analysis of NF-κB activity

NF-κB p65 concentration was quantified to assess NF-κB activity in *in vitro* and *in vivo*. Activated NF-κB p65 in the nucleus was extracted from GBC cells or excised tumors using a nuclear extraction kit (Active Motif, Carlsbad, CA, USA) and quantified with an enzyme-linked immunosorbent assay (ELISA) kit (TransAM NF-κB p65; Active Motif). All procedures were performed according to the attached instruction manual.

### Western blotting

NOZ and OCUG-1 cells were seeded (1 × 10^6^ cells/100-mm dish and 4 × 10^5^ cells/100-mm dish, respectively) and subjected to each treatment regimen for 36 and 72 h, respectively, *in vitro*. The protocol for western blotting has been described previously [19]. After overnight incubation with each primary antibody (1:1000 dilution) at 4°C, the membranes were incubated with secondary antibody (1:10,000 dilution, Histofine; Nichirei, Tokyo, Japan) for 2 h at room temperature. The protein bands were detected by using Clarity Max Western ECL Substrate (BIO-RAD, Hercules, CA, USA), a Chemi Doc XRS+ system, and the Image Lab Software (BIO-RAD). The results were quantified by densitometry and normalized to β-actin expression.

### Annexin V-FITC assay

GBC cells were seeded (2 × 10^5^ cells/100-mm dish) and subjected to each treatment regimen for 96 h. The induction of apoptosis by each treatment was assessed using a MEBCYTO Apoptosis Kit (Annexin V-FITC Kit; MBL, Nagoya, Japan). The treated cells were stained with a combination of Annexin V-FITC and propidium iodide. Apoptotic cells were detected by an Attune NxT Flow Cytometer (Thermo Fisher Scientific, Waltham, MA, USA). All procedures were performed according to the instruction manual.

### Cell cycle analysis

NOZ and OCUG-1 cells were seeded (1 × 10^6^ cells/100-mm dish) and subjected to each treatment regimen for 36 and 72 h, respectively. GBC cells were fixed in 70% ethanol for 30 minutes at -20°C, and the centrifuged cell pellets were washed with PBS. Finally, these cells were incubated with 0.5 ml PI/RNase Staining Buffer (BD Biosciences, Franklin, NJ, USA) for 15 minutes at room temperature. DNA content was analyzed with an Attune NxT Flow Cytometer (Thermo Fisher Scientific).

### Wound healing assay

NOZ and OCUG-1 cells were seeded in 6-well plates (5 × 10^5^ cells per well) to form a confluent monolayer. After culturing for 24 h, a sterile 1,000 μL pipette tip was used to scratch the monolayer in all wells. The cells in each well were then treated as described. The distance between cells was taken at the same position at 0 and 24 h using an IX71-Olympus inverted microscope (Olympus, Tokyo, Japan) at 10 × magnification. The wound healing index is expressed as a percentage of the wound area at 24 h divided by that at 0 h.

### Invasion assay

The invasive ability of GBC cells was assessed using a CytoSelect^TM^ 96-well Cell Invasion Assay kit (Cell Biolabs, San Diego, CA, USA). After irradiating GBC cells with 5 Gy, NOZ and OCUG-1 cells were seeded into 96-well migration plate (1 × 10^5^ and 5 × 10^4^ cells per well, respectively), and incubated with NM (80 and 40 μg/mL, respectively). Invading cells were quantified by reading the fluorescence at 480nm/ 520nm by using a Synergy H1 Hybrid Multi-Mode Microplate Reader (BioTek, Winooski, VT, USA). All procedures were performed according to the instruction manual.

### Animals and *in vivo* experimental protocols

Five-week-old nude mice (BALBc nu/nu) were obtained from CLEA Japan, Inc. (Tokyo, Japan). All mice were male to eliminate gender bias. The mice were raised under specific pathogen-free conditions at the Laboratory Animal Facility of the Jikei University School of Medicine.

A xenograft GBC mouse model was established by subcutaneous injection of 5 × 10^6^ NOZ cells into the right flank. At one week after subcutaneous inoculation, the mice were assigned to the following groups: (1) NM group, intraperitoneal (i.p.) injection of NM (30 mg/kg) five times a week (n = 20); (2) radiation group, radiation (5 Gy) once and i.p. injection of DW (30 mg/kg) five times a week (n = 16); (3) combination group, radiation (5 Gy) once and i.p. injection of NM (30 mg/kg) five times a week (n = 17); and (4) control group, i.p. injection of DW (30 mg/kg) five times a week (n = 20). The treatments were administered for two weeks. We measured the diameter of the subcutaneous tumors and body weight three times a week during the treatment period. The animals were sacrificed at two weeks after the start of the treatment to ensure that the diameter of the subcutaneous tumors did not exceed 20 mm. The tumors were removed after sacrifice, fixed with 4% paraformaldehyde, and embedded in paraffin for immunohistochemistry. The mice were euthanized if significant weakness was observed by the time of sacrifice (e.g. the tumor weighs more than 10% of body weight; weight loss of 20% or more in 2–3 days, or 25% or more in 7 days). All surgery was performed under isoflurane anesthesia, and all efforts were made to minimize suffering. This study was carried out in strict accordance with the recommendations in the Guide for the Care and Use of Laboratory Animals of the National Institutes of Health. The protocols for all animal experiments were approved by the Institutional Animal Care and Use Committee of the Jikei University School of Medicine (Protocol number: 2017–052).

### Immunohistochemistry

Sections sliced from paraffin-embedded excised tumor tissues were immunohistochemically stained using anti-Ki-67 antibodies (1:300 dilution) and visualized using an Invitrogen EVOS FL Auto 2 Imaging System (Thermo Fisher Scientific, Waltham, MA, USA). The percentage of Ki-67-positive cells was microscopically examined in three random high-power fields at 400× in all excised tumors.

### Statistical analyses

All data were statistically analyzed with SPSS 25.0 (IBM Japan, Tokyo, Japan) and expressed as a mean ± standard deviation. The non-paired *t-*test (two-tailed) and one-way analysis of variance were used for the analyses. Results with p values < 0.05 were considered statistically significant.

## Results

### Radiotherapy and NM suppresses the viability of GBC cells

**Fig 1:**
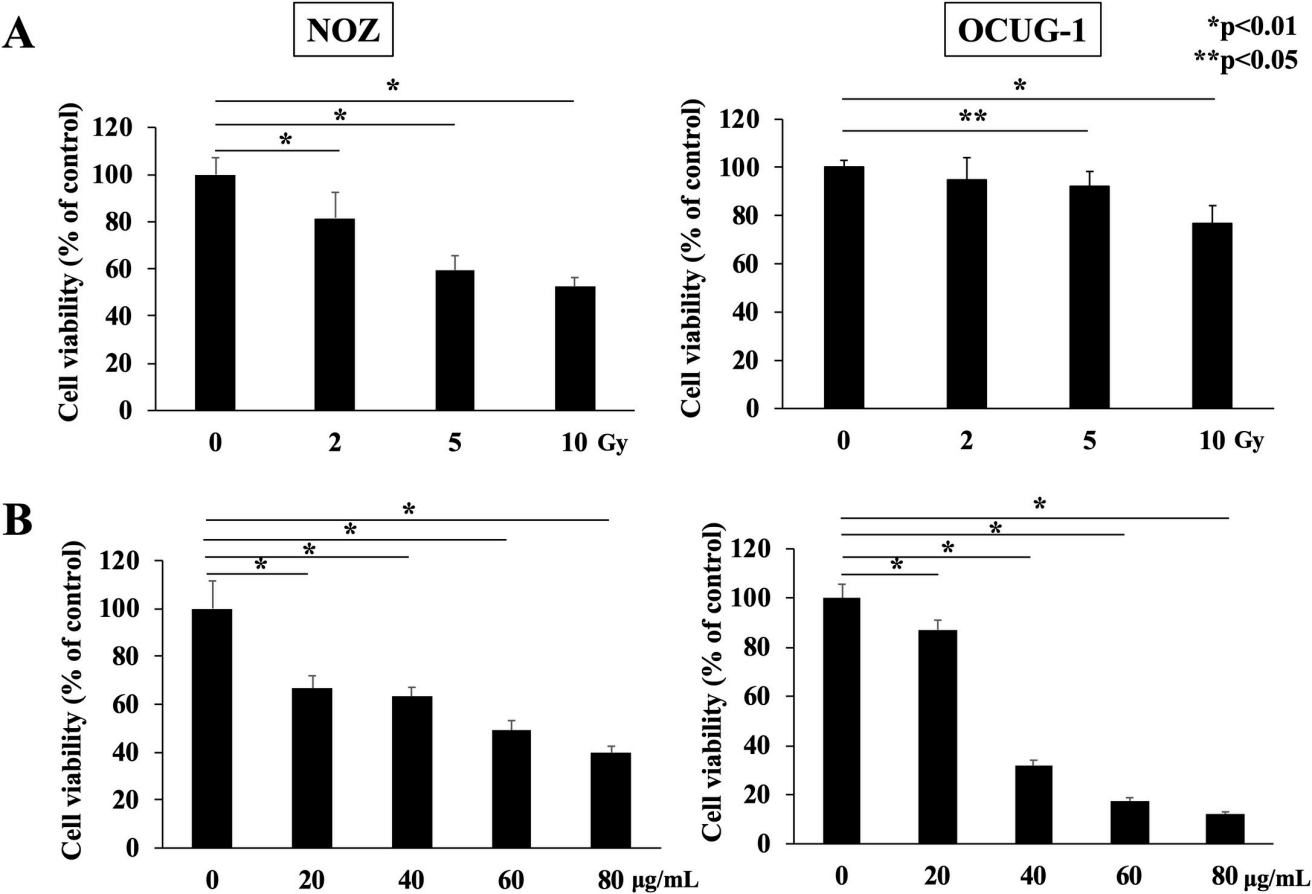
Cell viabilities of NOZ and OCUG-1 cells treated with radiotherapy or NM. (A) Cell viability was suppressed by radiotherapy in a dose-dependent manner (NOZ: 0 Gy: 100.0 ± 7.1%; 2 Gy: 81.7 ± 10.8%; 5 Gy: 59.5 ± 6.1%; 10 Gy: 52.7 ± 3.7%, OCUG-1: 0 Gy: 100.0 ± 3.0%; 2 Gy: 94.6 ± 9.5%; 5 Gy: 92.0 ± 6.4%; 10 Gy: 77.1 ± 7.1%) at 96 h after irradiation. (B) Cell viability was suppressed by NM in a dose-dependent manner (NOZ: 0 μg/mL: 100.0 ± 11.5%; 20 μg/mL: 66.7 ± 5.2%; 40 μg/mL: 63.3 ± 3.8%; 60 μg/mL: 49.2 ± 4.0%; 80 μg/mL: 39.6 ± 2.9%, OCUG-1: 0 μg/mL: 100.0 ± 5.6%; 20 μg/mL: 87.7 ± 3.9%; 40 μg/mL: 31.8 ± 2.2%; 60 μg/mL: 17.4 ± 1.3%; 80 μg/mL: 12.2 ± 0.8%).

To determine the adequate irradiation dose to achieve a cytotoxic effect, the viabilities of NOZ and OCUG-1 cells at each radiation dose (2, 5, and 10 Gy) were measured using the WST-1 assay. The viabilities of both cell lines were suppressed in a radiation dose-dependent manner (Fig 1A). Although 5 Gy of radiation decreased NOZ cell proliferation by 40%, it inhibited OCUG-1 cell proliferation by only 10%. These results indicated that OCUG-1 cells were potentially more radio-resistant than NOZ cells. The same tendency was observed in the colony forming assay (S1 Fig).

Next, to evaluate the effect of NM dose, the cell viabilities of NOZ and OCUG-1 cells after treatment with each NM dose were measured. The viabilities of both cell lines were suppressed in a NM dose-dependent manner (Fig 1B).

### NM inhibits radiation-induced NF-κB activity *in vitro*

**Fig 2:**
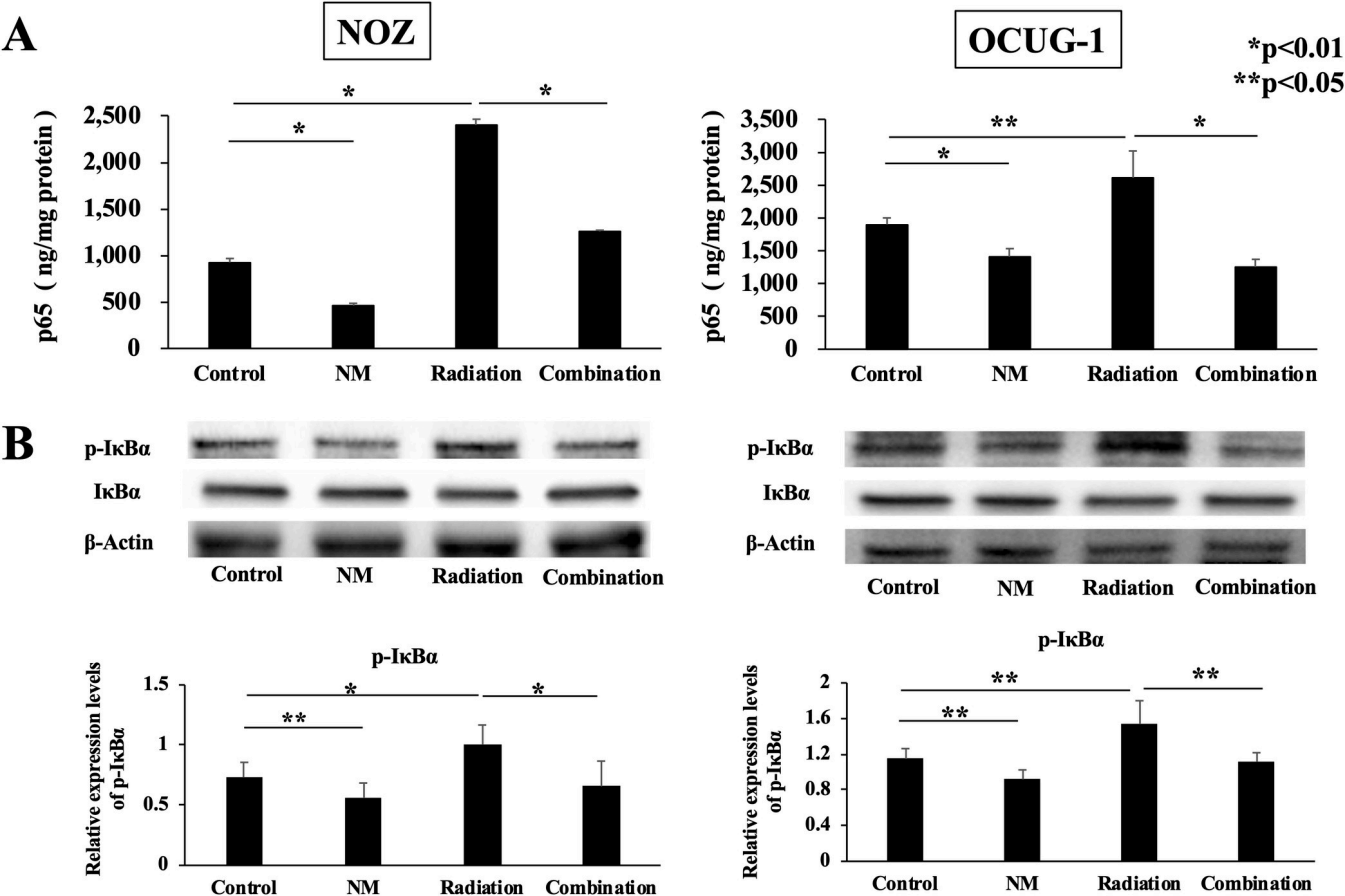
NF-κB p65 and phosphorylation of IκBα *in vitro*. (A) The enzyme-linked immunosorbent assay (ELISA) results showed that p65 concentration in the radiation group was higher than that in the control group (NOZ: 2,398.4 ± 62.8 ng/mg protein vs. 923.5 ± 42.3 ng/mg protein, p < 0.01; OCUG-1: 2,609.3 ± 409.3 ng/mg protein vs. 1,899.5 ± 99.5 ng/mg protein, p < 0.05). p65 concentration in the combination group was lower than that in the radiation group (NOZ: 1,256.1 ± 13.0 ng/mg protein vs. 2,398.4 ± 62.8 ng/mg protein, p < 0.01; OCUG-1: 1,250.9 ± 117.8 ng/mg protein vs. 2,609.3 ± 409.3 ng/mg protein, p < 0.01). (B) Western blotting results revealed that the expression level of phosphorylated IκBα decreased in the combination group compared with that in the radiation group (NOZ; p < 0.01, OCUG-1; p < 0.05), whereas the expression level of IκBα increased in the combination group compared with that in the radiation group at 3 h for NOZ and OCUG-1 cells.

To clarify the mechanism of radio-resistance in GBC cells, we quantified NF-κB in nuclear extracts. NF-κB was activated constitutively in NOZ and OCUG-1 cells, and radiotherapy potentiated NF-κB activity (NOZ; p < 0.01, OCUG-1; p < 0.05, Fig 2A). On the contrary, NM suppressed not only steady NF-κB activity but also radiation-induced NF-κB activity (NOZ; p<0.01, OCUG-1; p<0.01, Fig 2A). To elucidate the mechanism of NF-κB inactivity by NM, phosphorylated IκBα (p-IκBα) was measured by western blotting using whole-cell extracts. The results showed that NM reduced the expression of p-IκBα, which indicated that NM blocked the transfer of NF-κB from the cytoplasm to the nucleus by blocking IκBα phosphorylation (Fig 2B).

### NM enhances cell apoptosis

**Fig 3:**
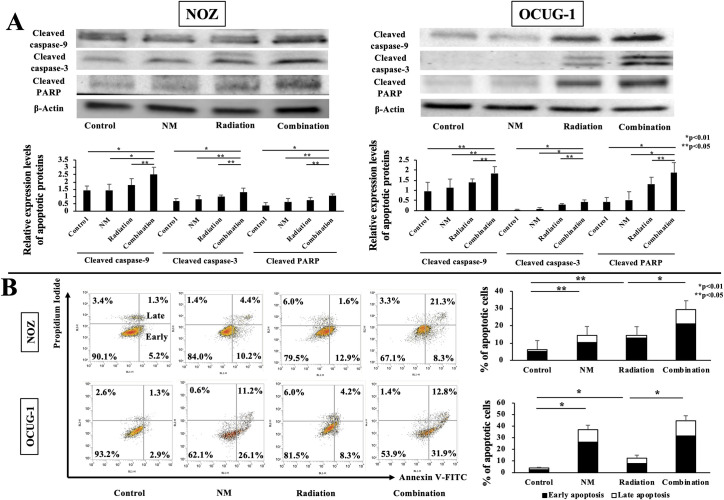
Quantification of apoptosis and apoptotic signals *in vitro*. (A) Western blotting results revealed that the expression levels of cleaved caspase-9, -3, and PARP in the combination group were higher than those in the other groups. (B) Annexin V/ FITC analysis by flow cytometry revealed that the proportions of early, late, and total apoptotic cells in the combination group were higher than those in the radiation group for NOZ and OCUG-1 cells (NOZ: early apoptosis: 21.3 ± 5.2% vs. 12.9 ± 1.9%, p < 0.05; late apoptosis: 8.3 ± 0.9% vs. 1.6 ± 0.2%, p < 0.01; total apoptosis: 29.5 ± 6.1% vs. 14.5 ± 2.1%, p < 0.01; OCUG-1: early apoptosis: 31.9 ± 2.3% vs. 8.3 ± 2.0%, p < 0.01; late apoptosis: 12.8 ± 2.2% vs. 4.2 ± 0.8%, p < 0.01; total apoptosis: 44.7 ± 4.4% vs. 12.5 ± 2.7%, p < 0.01).

Activated NF-κB regulates gene transcription of antiapoptotic proteins and subsequently inhibits cell apoptosis [10, 13–15]. To evaluate the correlation between inhibition of NF-κB activity caused by NM and cell apoptosis, the induction of the caspase cascade (cleaved caspase-9, -3, and PARP) was examined by western blotting. In both cell lines, the expression levels of cleaved caspase-9, -3, and PARP after the combination treatment were higher than those after the other treatments (Fig 3A). Subsequently, we analyzed cell apoptosis by the Annexin V assay. In NOZ cells, radiotherapy and NM significantly induced cell apoptosis. Furthermore, combination treatment with radiation and NM enhanced apoptosis caused by NM alone, suggesting that these two treatments exerted a beneficial effect on apoptosis (p < 0.01, Fig 3B). However, although radiotherapy and NM significantly induced apoptosis in OCUG-1 cells, apoptosis induction by radiotherapy was inferior to that by NM. In the combination treatment group, cell apoptosis increased compared to that in the other three groups (Fig 3B).

### NM enhanced G2/M phase cell cycle arrest

**Fig 4:**
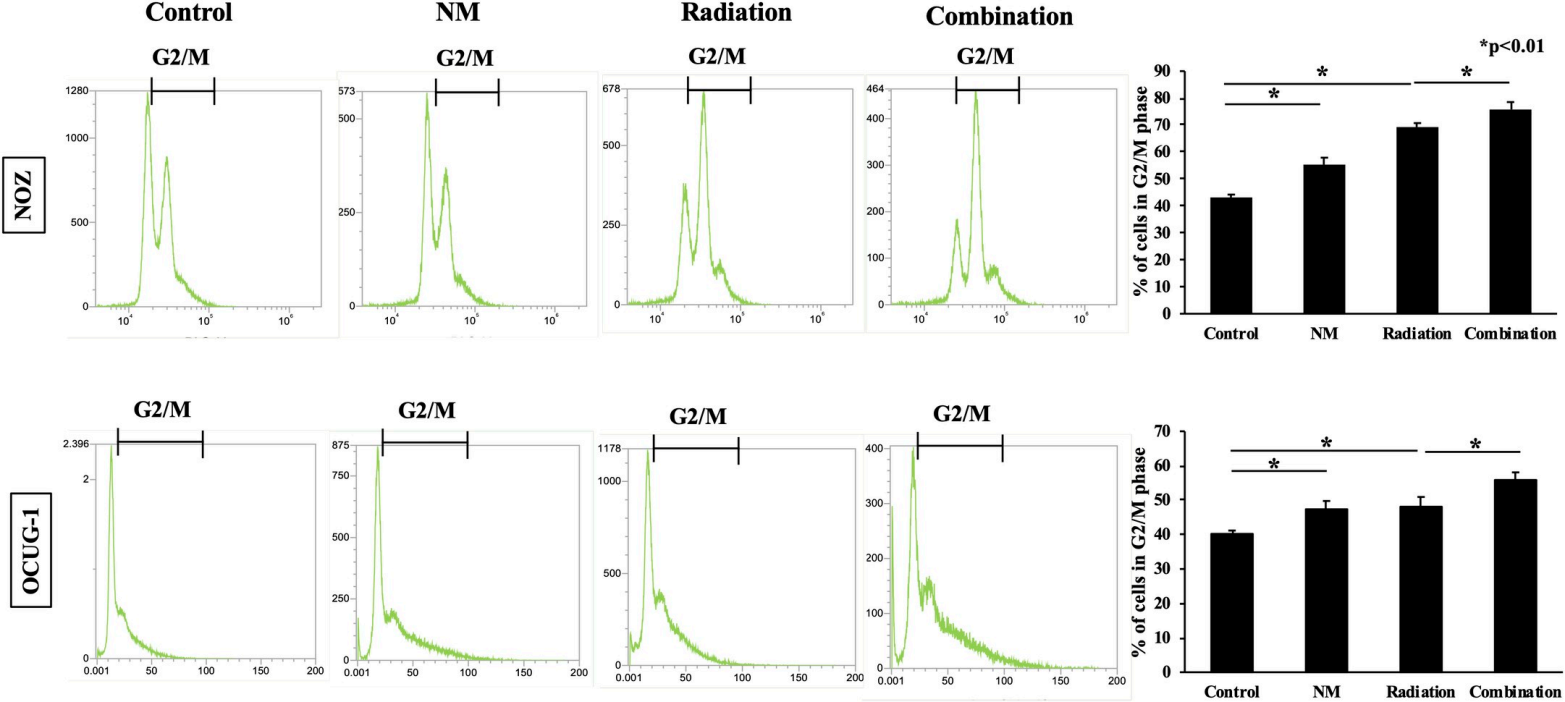
NM enhanced radiation-induced G2/M phase cell cycle arrest. Cell cycle analysis by flow cytometry revealed that the proportion of cells in G2/M phase in the combination group was higher than that in the radiation group for NOZ and OCUG-1 cells (NOZ: 75.8 ± 2.75% vs. 69.3 ± 1.5%, p < 0.01; OCUG-1: 55.9 ± 2.2% vs. 48.1 ± 2.8%, p < 0.01).

To evaluate the effect of NM on cell cycle progression, we investigated the distribution of the cell cycle in both cell lines. The proportion of cells in G2/M phase which indicated cell cycle arrest in the NM group and radiation group was higher than that in the control group (Fig 4). In addition, the proportion of G2/M phase cells in the combination group was higher than that in the radiation group (Fig 4).

### NM suppressed the migration and invasion of GBC cells

**Fig 5:**
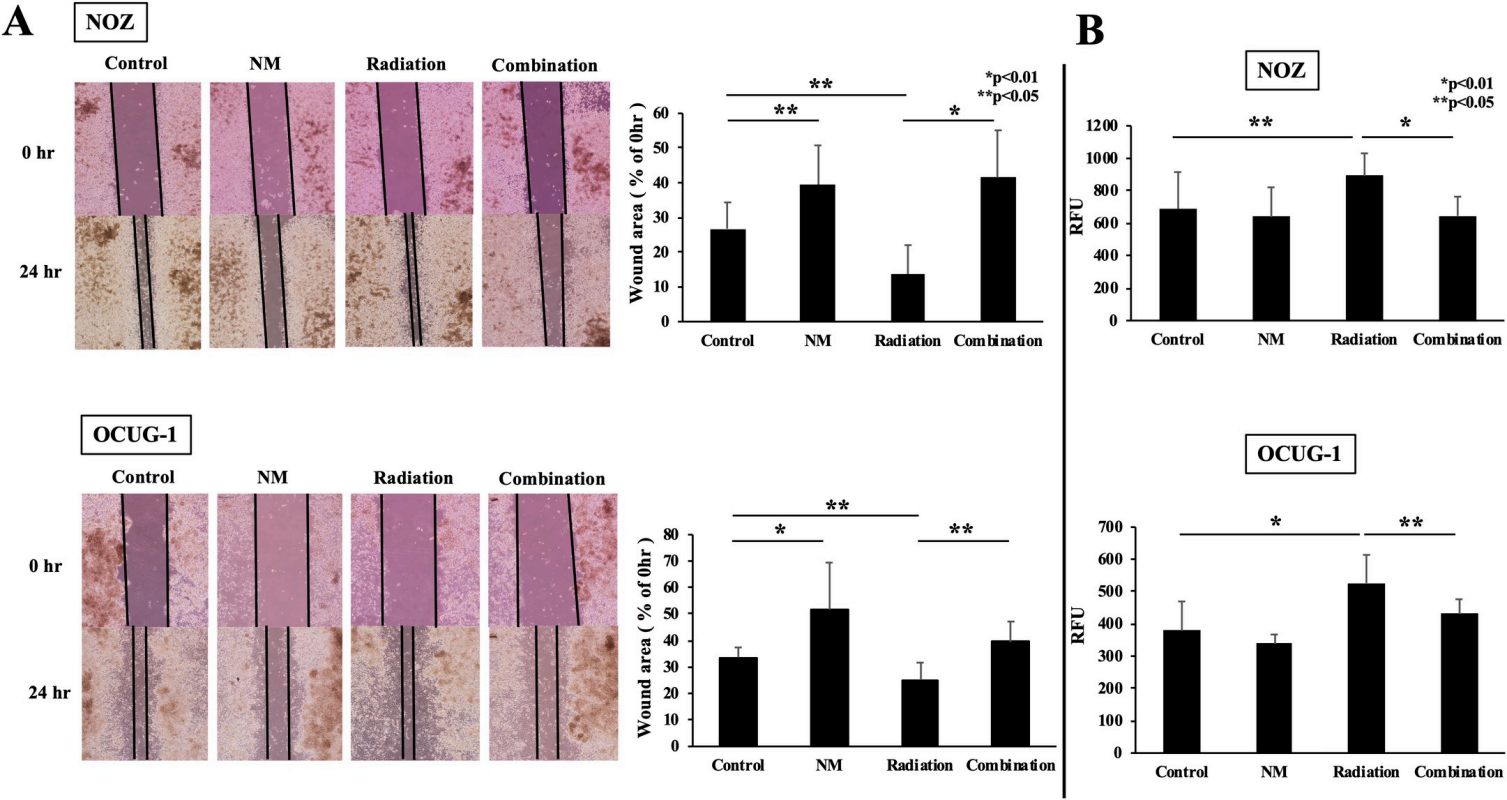
NM suppressed radiation-induced migration and invasion of GBC cells. (A) The wound area in the combination group was wider than in the radiation group for NOZ and OCUG-1 cells (NOZ: 41.5 ± 13.6% vs. 13.7 ± 8.3%, p < 0.01; OCUG-1: 39.4 ± 7.7% vs. 25.1 ± 6.5%, p < 0.05). (B) The invasion index in the combination group was lower than in the radiation group for NOZ and OCUG-1 cells (NOZ: 644.7 ± 118.6 vs. 891.1 ± 139.2, p < 0.01; OCUG-1: 430.6 ± 45.9 vs. 526.4 ± 87.5, p < 0.05). RFU: Relative fluorescence units.

To investigate whether NM suppressed radiation-induced migration and invasion of GBC cells, we performed wound healing assay and invasion assay. In wound healing assay, the wound area in the radiation group was narrower than that in the control group in both cell lines (Fig 5A). This result suggested that radiotherapy enhanced migration abilities of cancer cells. On the contrary, NM suppressed radiation-induced migration (Fig 5A). Subsequently, the effect of NM on cancer invasiveness was evaluated. In invasion assay, radiotherapy also potentiated the invasive ability in both cells (Fig 5B). In addition, the invasion index in the combination group was lower than that in the radiation group (Fig 5B). These results indicated that NM suppressed radiation-induced migration and invasion in GBC cells.

### NM improves radio-resistance of GBC cells

**Fig 6:**
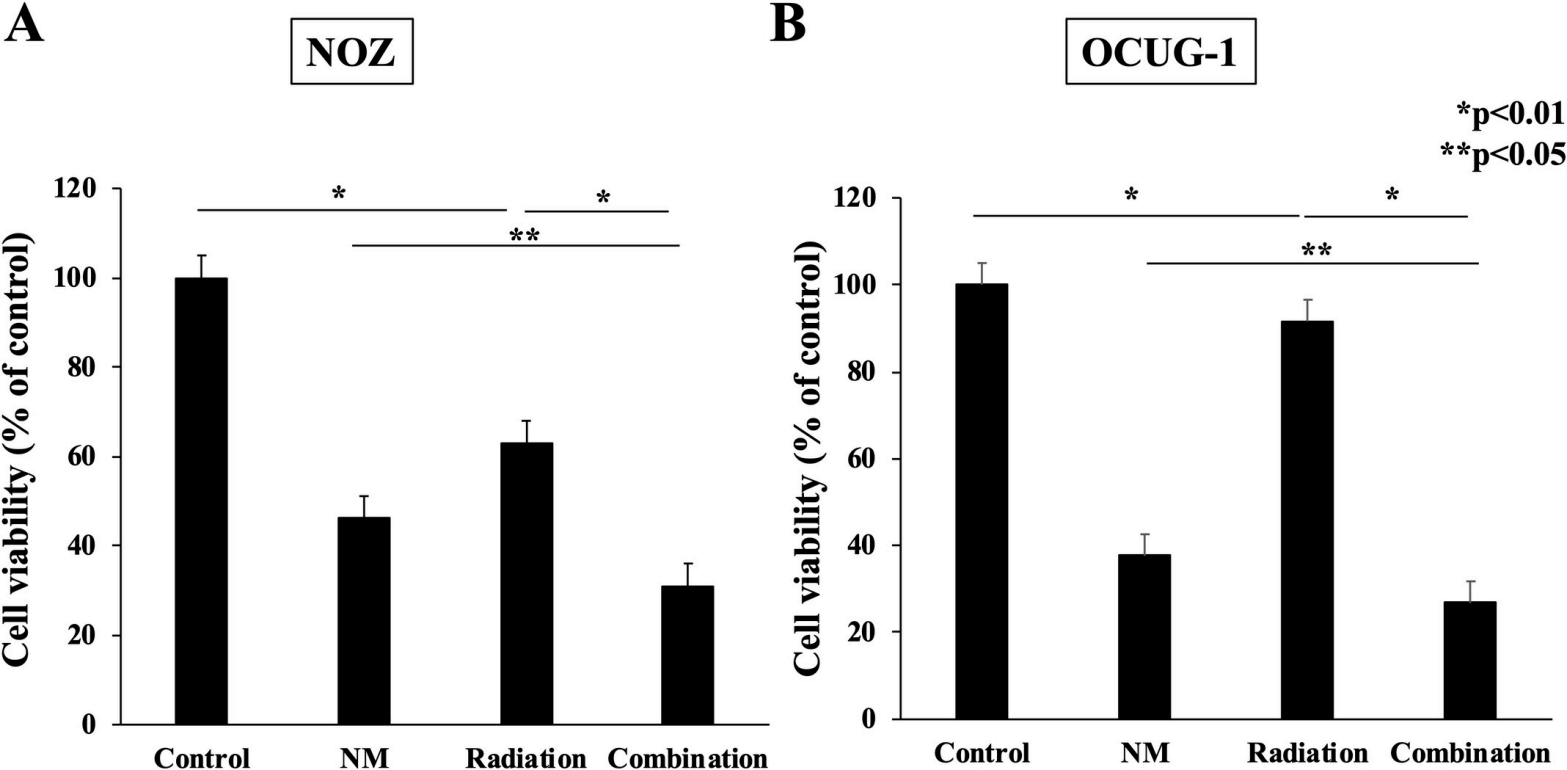
Cell proliferation evaluated using WST-1 assay. Cell viability in the combination group was suppressed compared with that in the radiation group for NOZ and OCUG-1 cells (NOZ: 31.1 ± 6.5% vs. 63.0 ± 4.0%, p < 0.01; OCUG-1: 26.8 ± 5.5% vs. 91.6 ± 2.2%, p < 0.01).

To assess the antitumor actions of NM, we examined the viability of GBC cells in each treatment group. In NOZ cells, NM administration increased the antiproliferative effect of radiation from 40% to 70% (p < 0.01, Fig 6A). In OCUG-1 cells, although radiation monotherapy decreased cell proliferation by approximately 10%, NM potentiated the antiproliferative effect of radiation to almost 70% (p < 0.01, Fig 6B). These results indicated that NM suppressed radiation-induced IκBα phosphorylation and NF-κB activity, which elevated the expression levels of apoptosis-related proteins and induction of cell apoptosis. Further, NM improved cell resistance to radiotherapy and enhanced the antiproliferative effects of radiotherapy in NOZ and OCUG-1 cells. In addition, we performed a colony forming assay for GBC cells treated with radiotherapy with or without NM. Colony forming assay showed that almost no colonization was observed in the group using NM (S2 Fig).

### NM suppresses tumor growth *in vivo*

**Fig 7:**
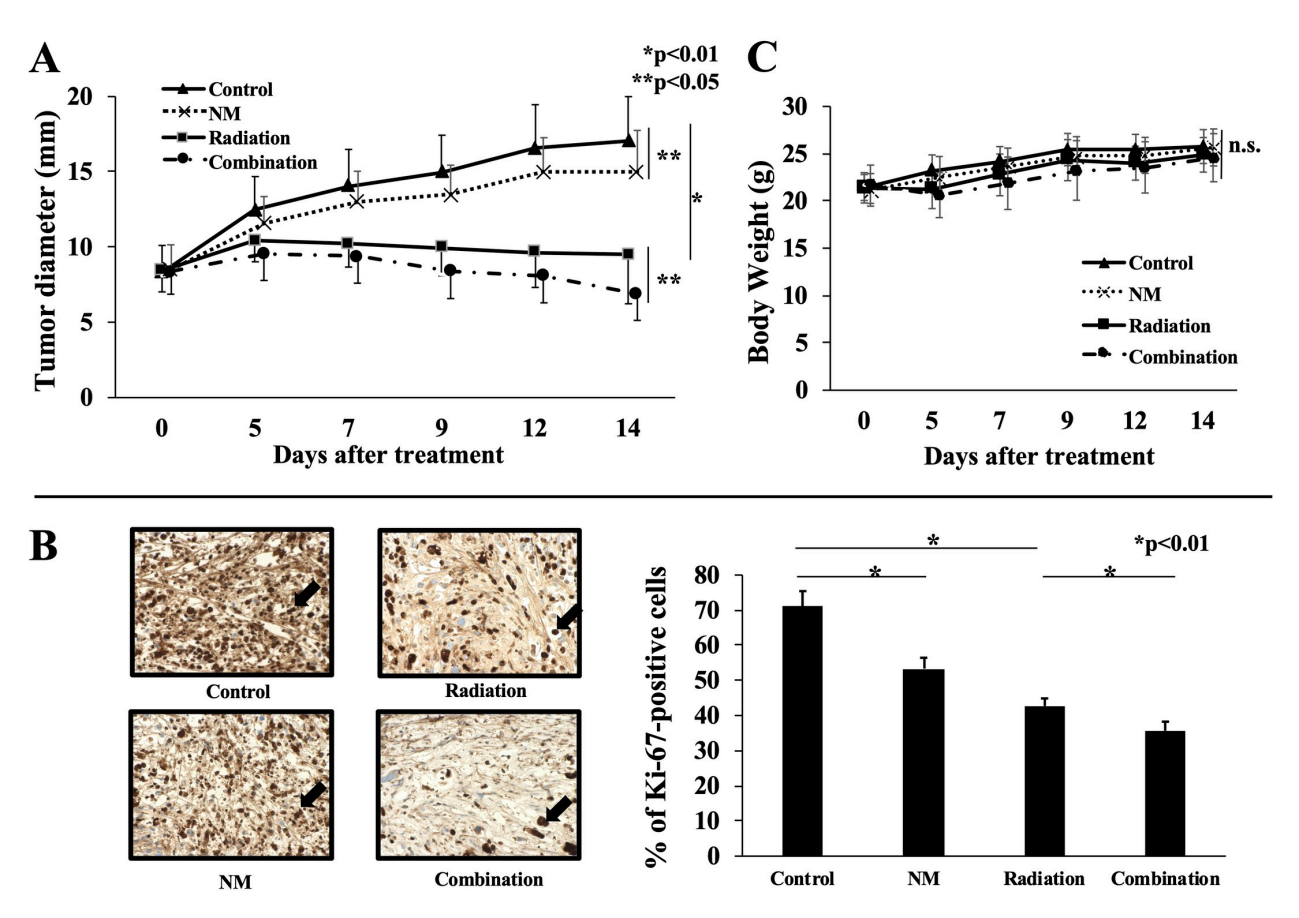
Growth of subcutaneous tumors in xenograft GBC mouse models. (A) At two weeks after treatment, the tumor diameter in the combination group was smaller than that in the radiation group (6.7 ± 1.8 mm vs. 9.5 ± 3.3 mm, p < 0.05). In addition, the tumor diameter in the NM group was suppressed compared with that in the control group (14.8 ± 2.8 mm vs. 17.1 ± 2.9 mm, p < 0.05). (B) Immunohistochemical staining showed that the percentage of Ki-67-positive cells in the combination group was lower than that in the radiation group (35.7 ± 2.6% vs. 42.7 ± 2.2%, p < 0.01). Ki-67-positive cells are indicated with arrows (400×). (C) Body weights of mice in the four groups were comparable during the treatment period.

A GBC xenograft mouse model was established to determine the antitumor actions of radiotherapy combined with NM *in vivo*. Both radiation and NM monotherapy suppressed tumor growth. Furthermore, NM potentiated the antitumor action of radiation (p < 0.05, Fig 7A). Surprisingly, in the combination group, the tumor diameter was smaller after than before the treatment (p = 0.01, Fig 7A). In addition, we counted the number of Ki-67-positive cells in resected tumors by immunohistochemical staining. The number of Ki-67-positive cells decreased after radiotherapy and NM monotherapy (p < 0.01, Fig 7B). Furthermore, the combination group had fewer Ki-67-positive cells than did the radiation group (Fig 7B), which suggested that NM also potentiated the antiproliferative effect of radiotherapy *in vivo*. During the treatment period, we measured the body weights of mice to examine the side effects of the treatments. However, there were no differences in body weight between the four groups (Fig 7C).

### NM inhibits radiation-induced NF-κB activity and induces apoptosis of GBC cells *in vivo*

**Fig 8:**
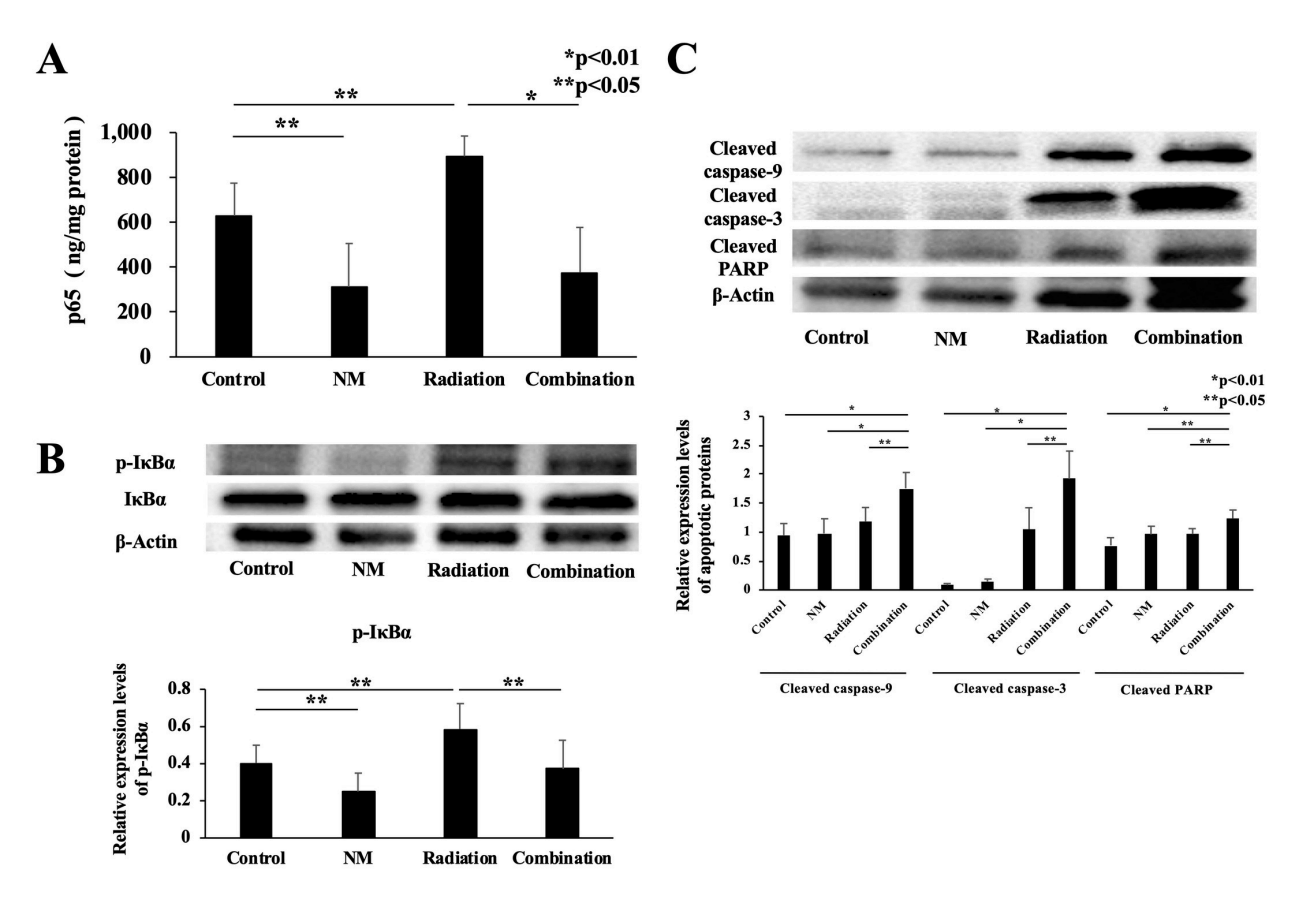
NF-κB and apoptotic signals *in vivo*. (A) p65 concentration in the excised tumors significantly increased in the radiation group compared with that in the control group (893.6 ± 91.0 ng/mg protein vs. 628.5 ± 146.1 ng/mg protein, p < 0.05). p65 expression in the combination group was lower than that in the radiation group (375.93 ± 201.3 ng/mg protein vs. 893.6 ± 91.0 ng/mg protein, p < 0.01). (B) Western blotting results revealed that the expression level of phosphorylated IκBα decreased in the combination group compared with that in the radiation group (p < 0.05). (C) The expression levels of apoptotic proteins in the combination group were higher than those in the other groups.

We assessed NF-κB activity and the expression levels of apoptosis-related proteins in the resected tumor. NM attenuated NF-κB activity by inhibiting radiotherapy-induced IκBα phosphorylation (Fig 8A and 8B). Furthermore, the expression levels of apoptosis-related proteins in xenograft tumors were the highest in the combination group (Fig 8C). The results were similar to those observed *in vitro*.

## Discussion

According to the National Comprehensive Cancer Network Guidelines, unresectable GBC is classified as locally advanced GBC and metastatic GBC [20]. For locally advanced GBC, chemotherapy using gemcitabine with cisplatin is recommended [20]. Radiotherapy is often considered as a symptomatic treatment for unresectable locally advanced GBC. On the contrary, some studies have reported that radiotherapy is useful as a perioperative treatment. However, large-scale randomized controlled trials on the effect of radiation treatment have not been realized [4–7]. The main mechanism underlying the antitumor action of radiotherapy involves induction of cell apoptosis via DNA double-strand break (DSB) [21, 22]. DNA DSB caused by ionizing radiation induces genotoxic stress [22]. In addition, DSB activates the IκB kinase-dependent NF-κB pathway, which causes treatment resistance. Therefore, we thought that NM-induced inhibition of NF-κB activity through blockade of IκBα phosphorylation would improve treatment resistance. We also focused on antiapoptotic mechanisms in the early and late stages of radiotherapy. Our results (Fig 3) showed that radiation monotherapy induced cell apoptosis. We hypothesized that radiation-induced NF-κB activity is correlated with the antiapoptotic mechanism. Moreover, NM inhibited radiation-induced IκBα phosphorylation and NF-κB activity (Fig 2), as well as enhanced the apoptotic signaling pathway *in vitro*.

Our data revealed that OCUG-1 cells were more radio-resistant than NOZ cells (Fig 1A). This difference in radiosensitivity may be due to differences in constitutive NF-κB activity between NOZ cells and OCUG-1 cells (Fig 2A). Higher constitutive NF-κB activity may lead to more severe radio-resistance. On the contrary, the radiosensitivity of OCUG-1 cells was markedly improved by blocking NF-κB activity via NM administration.

Additionally, we evaluated the effects of the treatments on cell cycle progression, migration, and invasion. In the cell cycle progression, radiotherapy increased the proportion of cells in G2/M phase which indicated cell cycle arrest, and the combined use of NM further marked the effect (Fig 4). On the contrary, radiotherapy potentiated the migration and invasion of GBC cells, while the combined use of NM suppressed the effect (Fig 5). These results were similar to our previous reports [17, 18]. In the present study, we attributed that the mechanisms by which the cell proliferation of GBC cells was suppressed by the combination therapy were that NM comprehensively worked to induce apoptosis, cell cycle arrest, and to suppress migration and invasion.

The inhibitor of apoptosis proteins (IAPs) family (e.g. X-linked IAP, cellular IAP1, and cellular IAP2), controlled by NF-κB, strongly inhibit apoptosis by inhibiting activated caspase [23]. Hence, by inhibiting NF-κB activity, the expression of IAPs can be reduced and therapeutic efficacy on malignant tumors may be enhanced. We previously showed that NM suppressed chemotherapy-induced NF-κB activity and showed antitumor action in GBC [24]. In addition, NM exerted antitumor actions in pancreatic cancer and colon cancer by inhibiting radiation-induced NF-κB activity [17, 18].

Based on these results, we conducted a combination treatment with radiation and NM, and the results showed enhancement of the antitumor action of radiotherapy on GBC cells though inhibition of radiation-induced NF-κB activity. In the present study, there was a difference in cell behavior after treatment with each therapy *in vitro* and *in vivo*. Although NM appeared to be superior to radiotherapy in terms of antiproliferative effects *in vitro* (Fig 6), the opposite was observed *in vivo* (Fig 7A). The reason for this was that the half-life of NM following i.p. administration was too short for it to reach subcutaneous tumors *in vivo*. Therefore, in clinical, NM is continuously administered by intravenous for DIC to keep an effective blood concentration.

Some studies have shown that inhibitors of NF-κB (e.g. thalidomide, bortezomib, and curcumin) exert antitumor action on some types of cancer cells [25–27]. On the contrary, chemoradiotherapy sometimes resulted in serious adverse events. The fact that this combination treatment did not induce body weight loss in animals in the present study indicated that this treatment might not cause malnutrition. Moreover, in Japan, NM has been used clinically since 1986 as a therapeutic drug for pancreatitis and DIC without causing serious adverse events [28, 29].

In conclusion, the present study showed that NM enhanced radiation-induced cell cycle arrest and apoptosis by suppressing radiation-induced NF-κB activity and suppressed radiation-induced migration and invasion of GBC cells. The radiotherapy combined with NM possessed the potential to be a useful treatment for patients with unresectable GBC. For future clinical applications, a combination of NM/radiotherapy with chemotherapy might be indispensable.

## Supporting information

S1 Fig Colony forming assay for GBC cells treated with radiotherapy. Colony images (A) and surviving fraction (B) showed that the number of colonies were reduced by radiotherapy in a dose-dependent manner in both GBC cells.
(TIF)

S2 Fig Colony forming assay for GBC cells treated with radiotherapy with or without NM. Colony forming assay showed that almost no colonization was observed in the group using NM.
(TIF)

S1 Raw images (PDF)
