# Supplementary figures and images for: Nafamostat mesilate, a nuclear factor kappa B inhibitor, enhances the antitumor action of radiotherapy on gallbladder cancer cells
Source: PLoS One. 2021 Sep 2;16(9):e0257019. doi: 10.1371/journal.pone.0257019 (PMC8412321; doi:10.1371/journal.pone.0257019)

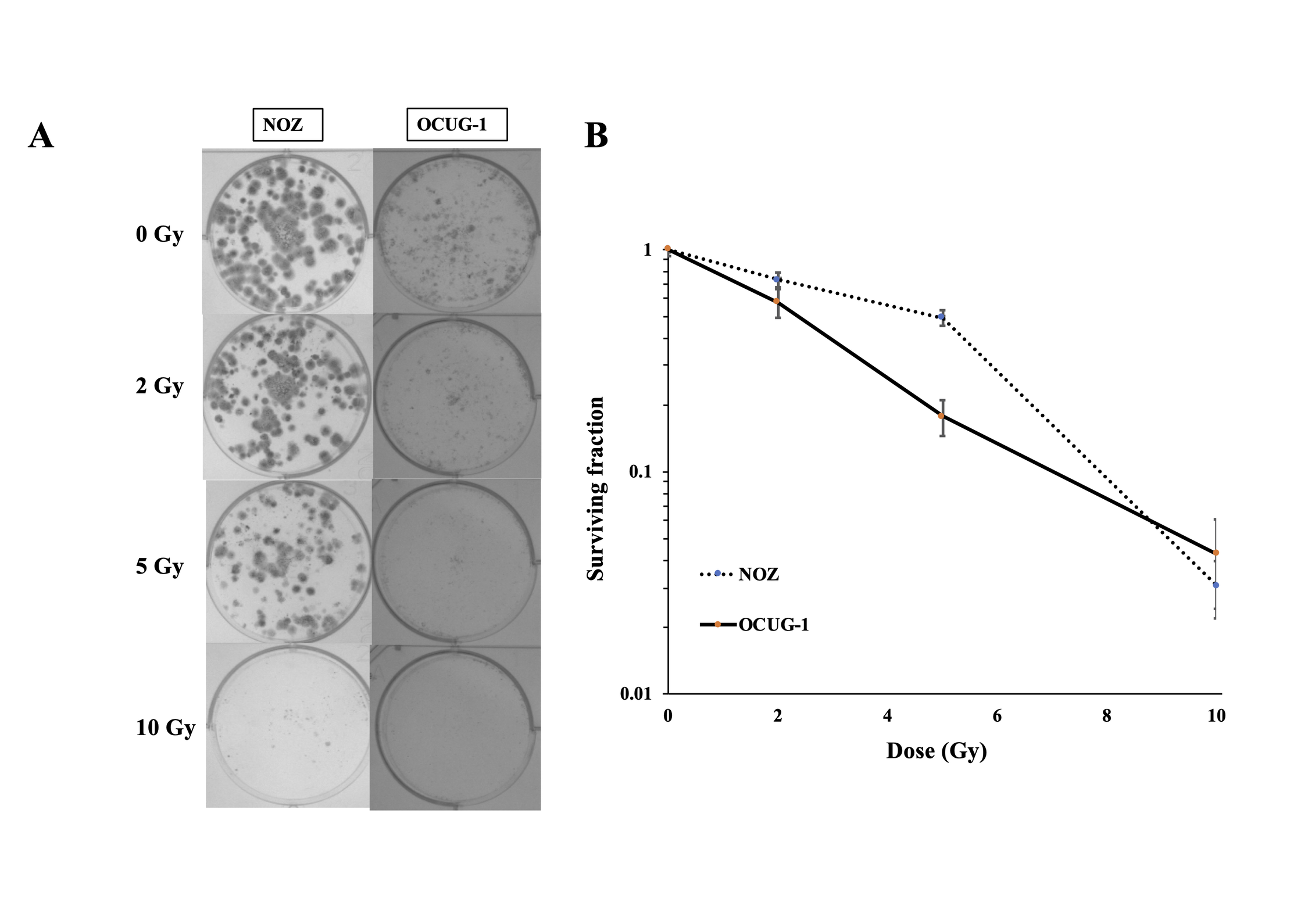

Supplement: S1 Fig — Colony images (A) and surviving fraction (B) showed that the number of colonies were reduced by radiotherapy in a dose-dependent manner in both GBC cells. (TIF) [file pone.0257019.s001.tif]

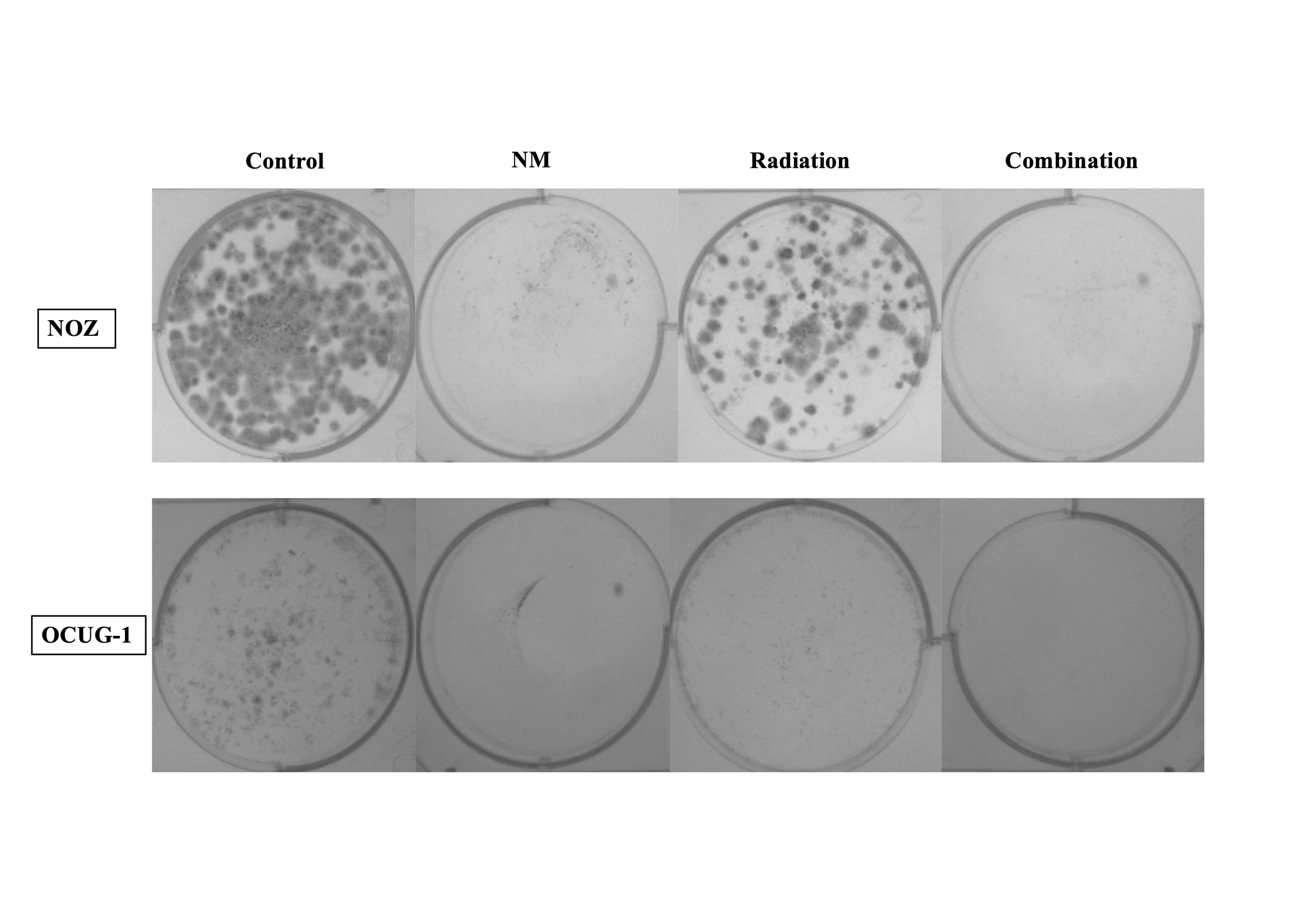

Supplement: S2 Fig — Colony forming assay showed that almost no colonization was observed in the group using NM. (TIF) [file pone.0257019.s002.tif]
