# Supplementary material for: Nafamostat mesilate, a nuclear factor kappa B inhibitor, enhances the antitumor action of radiotherapy on gallbladder cancer cells
Source: PLoS One. 2021 Sep 2;16(9):e0257019. doi: 10.1371/journal.pone.0257019 (PMC8412321; doi:10.1371/journal.pone.0257019)
Supplement: S1 Raw images — (PDF) [file pone.0257019.s003.pdf]

Fig 2B

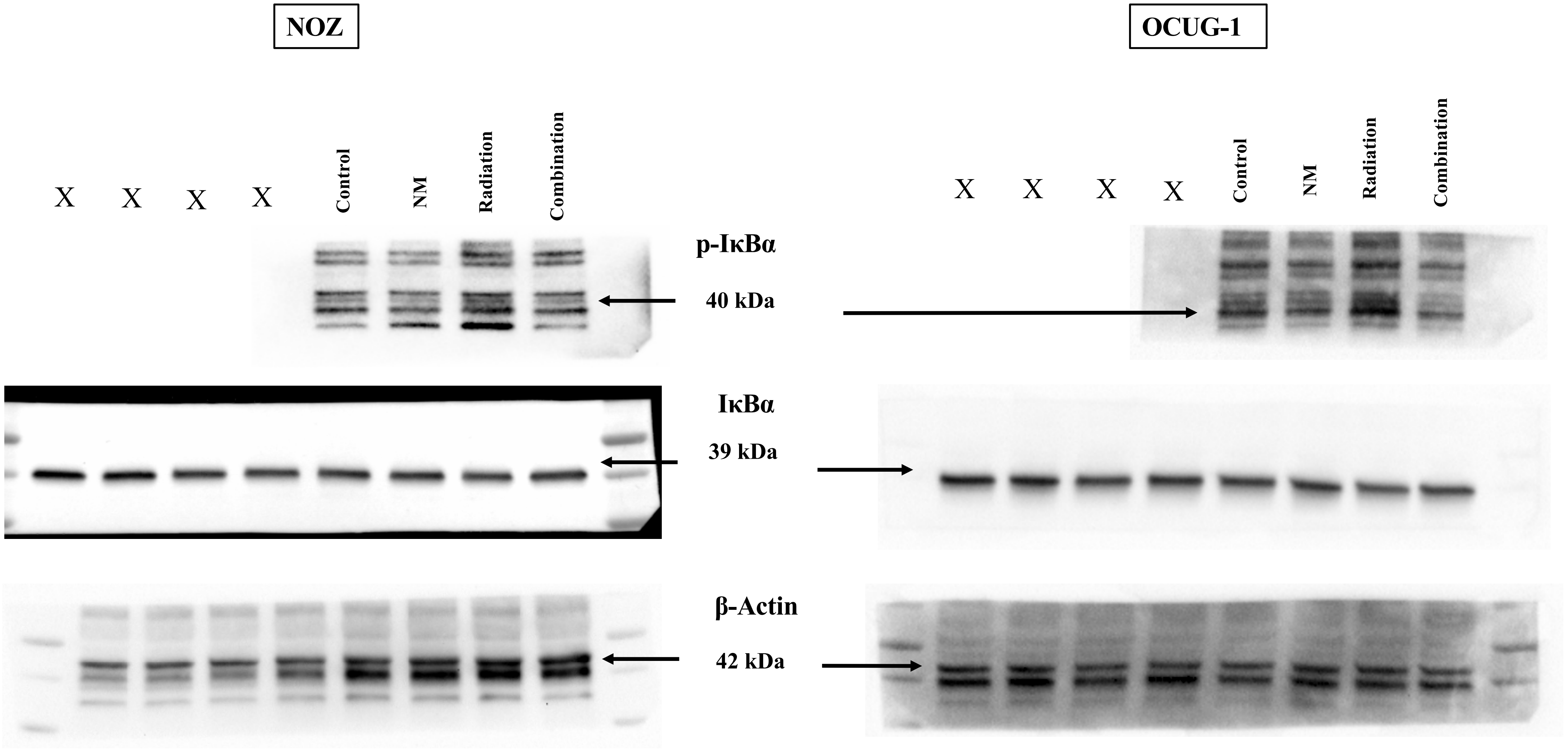
NOZ
OCUG-1
X
X
X
X
Control
NM
Radiation
Combination
p-IκBα
40 kDa
IκBα
39 kDa
β-Actin
42 kDa

Fig 3A

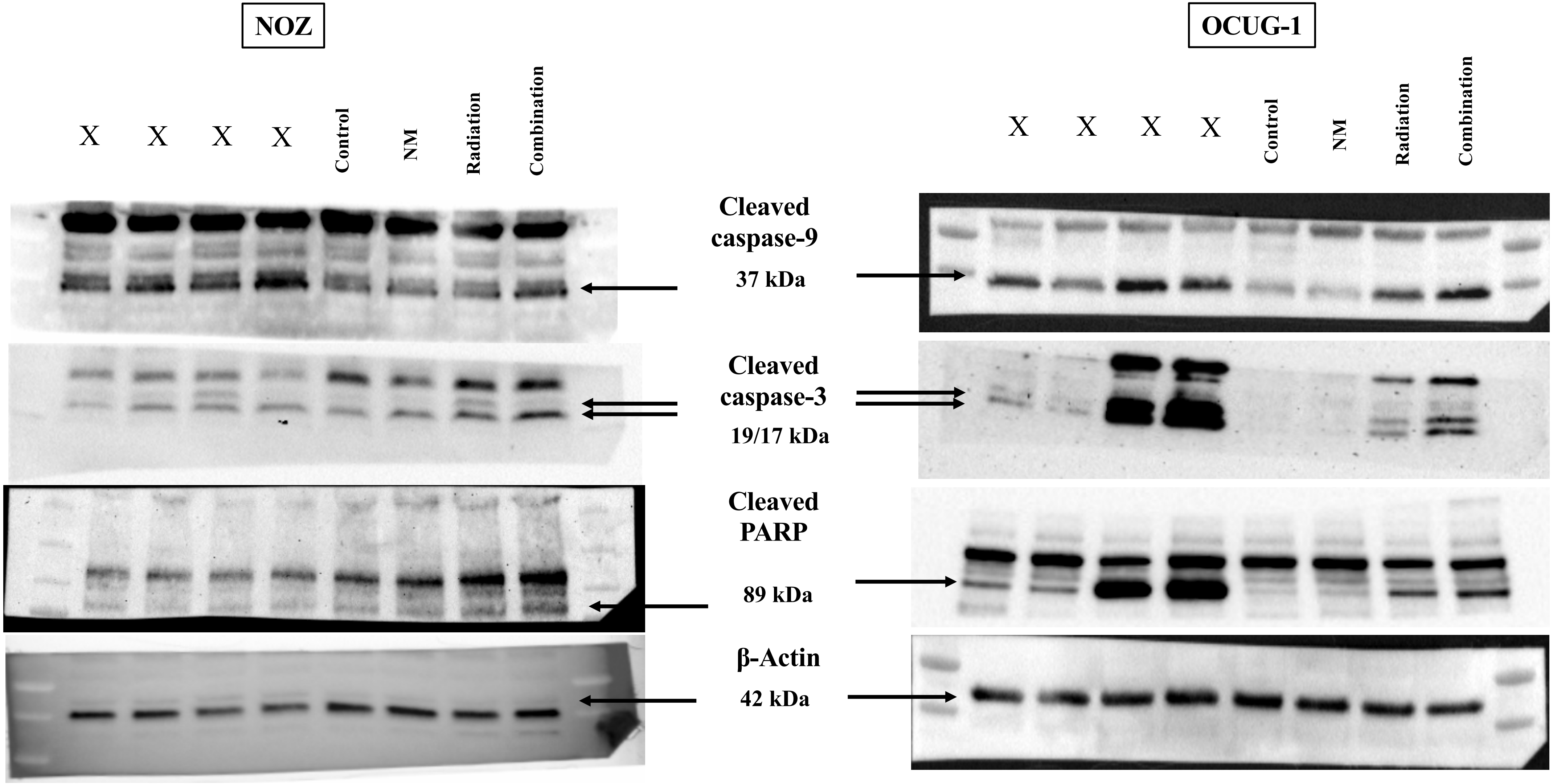

NOZ
OCUG-1
X
X
X
X
Control
NM
Radiation
Combination
Cleaved caspase-9
37 kDa
Cleaved caspase-3
19/17 kDa
Cleaved PARP
89 kDa
β-Actin
42 kDa

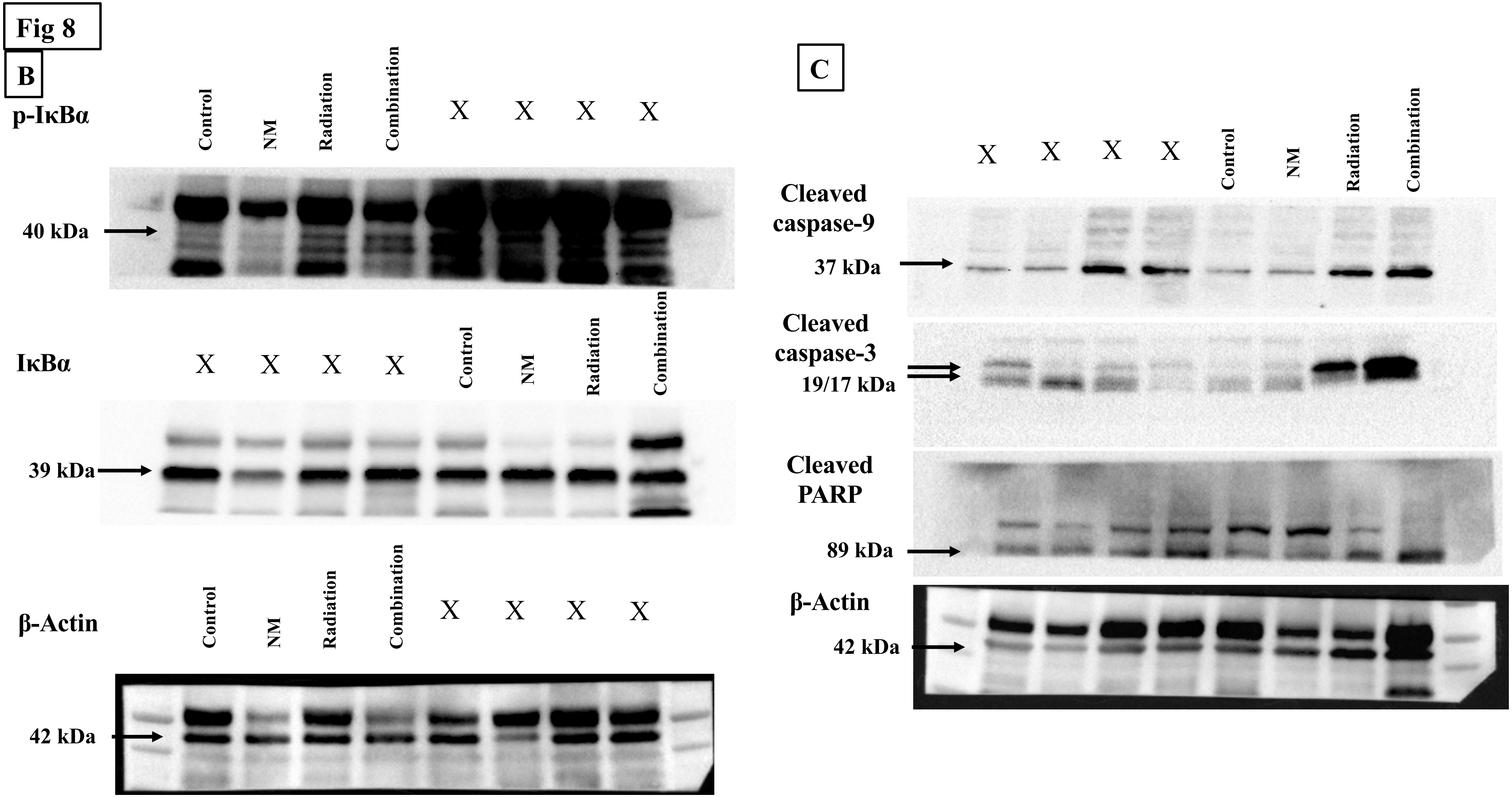

Fig 8
B
p-IκBα
Control
NM
Radiation
Combination
X
X
X
X
40 kDa
IκBα
X
X
X
X
Control
NM
Radiation
Combination
39 kDa
β-Actin
Control
NM
Radiation
Combination
X
X
X
X
42 kDa
C
X
X
X
X
Control
NM
Radiation
Combination
Cleaved caspase-9
37 kDa
Cleaved caspase-3
19/17 kDa
Cleaved PARP
89 kDa
β-Actin
42 kDa
